# Mucosal Immune System Dysregulation in the Pathogenesis of IgA Nephropathy

**DOI:** 10.3390/biomedicines10123027

**Published:** 2022-11-24

**Authors:** Toshiki Kano, Hitoshi Suzuki, Yuko Makita, Yoshihito Nihei, Yusuke Fukao, Maiko Nakayama, Mingfeng Lee, Rina Kato, Ryosuke Aoki, Koshi Yamada, Masahiro Muto, Yusuke Suzuki

**Affiliations:** 1 Department of Nephrology, Juntendo University Faculty of Medicine, Tokyo 113-8421, Japan; 2 Department of Nephrology, Juntendo University Urayasu Hospital, Chiba 279-0021, Japan

**Keywords:** IgA nephropathy, gut-associated lymphoid tissue, nasal-associated lymphoid tissue, galactose-deficient IgA1

## Abstract

The mucosal immune system, via a dynamic immune network, serves as the first line of defense against exogenous antigens. Mucosal immune system dysregulation is closely associated with the pathogenesis of immunoglobulin A nephropathy (IgAN), as illustrated by IgAN having the clinical feature of gross hematuria, often concurrent with mucosal infections. Notably, previous studies have demonstrated the efficacy of tonsillectomy and found that a targeted-release formulation of budesonide reduced proteinuria in patients with IgAN. However, it remains unclear how exogenous antigens interact with the mucosal immune system to induce or exacerbate IgAN. Thus, in this review, we focus on the dysregulation of mucosal immune response in the pathogenesis of IgAN.

## 1. Introduction

Immunoglobulin A nephropathy (IgAN) is characterized by the deposition of IgA and complement (C) 3 in the mesangial region. IgAN was first reported by Jean Berger and Hinglais in 1968 and accounts for 30%–50% of patients with primary glomerulonephritis (GN) [1,2]. According to long-term follow-up studies, 20%–40% of untreated patients develop end-stage kidney disease and require renal replacement therapy within 20 years [3,4]. The multi-hit theory was proposed regarding the development of IgAN [5]. Patients with IgAN have genetically increased levels of serum IgA1 that contains galactose-deficient *O*-glycans in its hinge region (galactose-deficient IgA1; Gd-IgA1) (Hit 1). Anti-glycan autoantibodies that recognize this Gd-IgA1 develop (Hit 2) and form pathogenic immune complexes (ICs) (Hit 3). These pathogenic ICs deposit in the glomeruli and activate mesangial cells, resulting in glomerular injury (Hit 4). However, the main site that produces Gd-IgA1 is still unknown.

A potential association between the mucosal immune system and IgAN has been proposed because the main production site of IgA is the mucosa and a common clinical feature of IgAN is gross hematuria, often concurrent with upper respiratory tract infection [6,7]. In support of this possibility, it was reported that the nephritis in IgAN-onset ddY mice was not exacerbated when these animals were housed in germ-free conditions, whereas, after stimulation with exogenous antigens, these mice exhibited mesangial IgA deposition with renal injury [8]. Furthermore, microbial infections have been found to influence various autoimmune responses and immunity [9,10]. The mucosa is constantly exposed to various exogenous antigens, and the mucosal immune system is important for their elimination. Mucosa-associated lymphoid tissue (MALT) is mainly divided into gut-associated lymphoid tissue (GALT) and nasal-associated lymphoid tissue (NALT), which have different roles and origins [11,12].

In this article, we will first review the current leading hypothesis on the pathogenesis of IgAN, and then we will focus on the mucosal immune response disorder centered on the GALT and NALT in IgAN.

## 2. Multi-Hit Mechanism of IgAN

IgA1 has an *O*-glycan in the hinge region between heavy-chain constant region domains 1 and 2. Allen and Hiki et al. reported that the IgA extracted from the renal glomeruli of patients with IgAN was mainly IgA1, in which the *O*-glycan side chains in the hinge region were highly under-glycosylated [13,14]. Their finding suggests that the reduced galactosylation and sialylation on the IgA1 hinge glycopeptide plays a pivotal role in the glomerular deposition of IgA. This scenario is further supported by the results of experiments conducted using KM55, an anti-Gd-IgA1 monoclonal antibody that we recently established [15]. The human α1 heavy chain contains several *O*-linked glycan chains, at positions 3–6, attached to the Serine and Threonine residues in the hinge region [16,17]. This glycosylation is performed stepwise by glycosyltransferases present in the Golgi apparatus of plasma cells that secrete IgA1, and diversity is observed in the *O*-glycan structures of IgA1 molecules [18]. Recently, it was found that Gd-IgA1 is associated with increased α-2,6-sialyltransferase 2 (ST6GalNAc-II) activity and decreased core 1 β1,3-galactosyltransferase (C1β3GalT) activity in patients with IgAN [19]. In addition, a portion of Gd-IgA1 derived from glomerular deposits in patients with IgAN is excreted in the urine. A recent report showed that the levels of urinary Gd-IgA1 were significantly higher in IgAN patients compared with disease controls [20]. Furthermore, levels of urinary Gd-IgA1 were significantly correlated with histopathological severity in IgAN patients, indicating that Gd-IgA1 plays a central role in the pathogenesis of IgAN [21].

In 70%–80% of patients with IgAN, their serum Gd-IgA1 levels are above the 90th percentile of levels in healthy controls [22]. Moreover, first-degree relatives of patients with IgAN exhibit elevated serum Gd-IgA1 levels comparable to patient levels, indicating that this condition has significant heritability [23]; however, because most of these relatives with aberrant IgA1 glycosylation were asymptomatic, it is difficult to conclude that increased Gd-IgA1 levels alone are enough to generate clinical IgAN. Thus, additional genetic or environmental cofactors may be required for the expression and development of IgAN [23,24]. Yanagihara et al. stimulated human mesangial cells, using IgA1 extracted from IgA myeloma patients, and found that only polymeric IgA1 or ICs, but not monomeric IgA1, activated the mesangium [25]. This finding suggests that the formation of ICs triggered by Gd-IgA1 contributes to IgAN exacerbation. However, the mechanisms of how the Gd-IgA1-specific IgG/IgA antibodies are produced and how the autoantibodies against Gd-IgA1 drive the formation of pathogenic ICs remain uncertain.

Tomana et al. reported that the reactivity of serum IgG extracted from patients with IgAN to IgA1 was significantly decreased by the removal of *O*-glycans from the IgA1 hinge region, indicating that IgG antibodies against nephritic IgA may recognize *O*-glycans in the hinge region [26]. Furthermore, Suzuki et al. generated Epstein–Barr Virus-immortalized IgG-secreting cells from IgAN patients and found that the secreted IgG formed glycan-dependent ICs with Gd-IgA1 [27]. Recently, Rizk et al. demonstrated the presence of IgG that exhibited specificity for Gd-IgA1 in the deposits in glomeruli of patients with IgAN, including IgG that is not detectable by routine immunofluorescence microscopy [28]. Collectively, these findings further support the possibility that Gd-IgA1-specific IgG autoantibodies have a pivotal role in the pathogenesis of IgAN.

**Figure 1:**
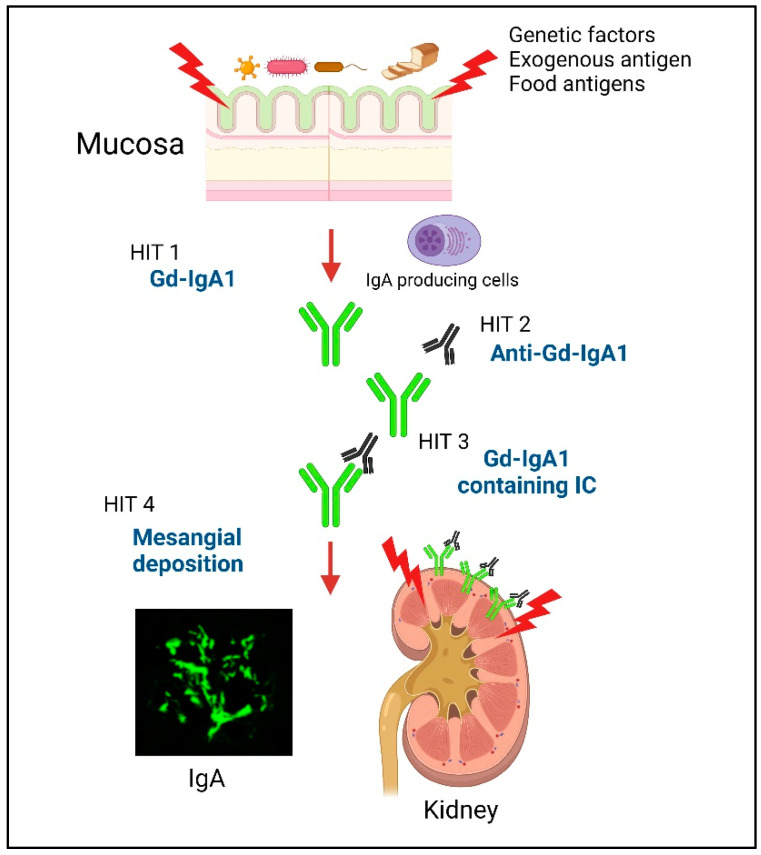
Schematic depicting the multi-hit model of IgAN development. Hit 1: Patients with IgAN have genetically increased levels of serum IgA1 that contains galactose-deficient *O*-glycans in its hinge region (galactose-deficient IgA1; Gd-IgA1). In addition, a disorder of the mucosal immune response against exogenous antigens in the MALT, causes an increase in the levels of Gd-IgA1 through Toll-like receptors (TLRs). Hits 2 and 3: Anti-glycan autoantibodies that recognize this Gd-IgA1 develop and form pathogenic immune complexes (ICs). Hit 4: These pathogenic ICs deposit in the glomeruli and activate mesangial cells, resulting in glomerular injury. Created with BioRender.com.

Previous work found that the injection of aberrantly glycosylated IgA from IgAN-onset mice into nude mice induced rapid IgA deposition along the glomerular capillary wall and in the mesangium [29]. Furthermore, we showed that the injection of in vitro-formed human Gd-IgA1–IgG ICs into nude mice resulted in the formation of glomerular immunodeposits and induced pathological changes similar to those associated with human IgAN [30]. In addition, Gd-IgA1-containing ICs induced glomerular endothelial cell activation in nude mice and caused the upregulation of chemokines, pro-inflammatory cytokines, and adhesion molecules, resulting in endothelial injury that alters the endothelial permeability, thus facilitating the entry of ICs into the mesangial region [30]. Together, these findings suggest that, in patients with IgAN, Gd-IgA1-containing ICs are continuously supplied to glomerular regions and deposited in the mesangium, resulting in the activation of mesangial cells and subsequent induction of glomerular injury. The multi-hit mechanism is summarized in Figure 1.

## 3. The Main Production Site of Gd-IgA1 in IgAN

Gd-IgA1 is recognized as a key molecule in the pathogenesis of IgAN; however, it is still unclear where it is produced. A connection between the mucosa-associated immune system and IgAN is indicated by the following: (1) a main clinical feature of IgAN is gross hematuria after an upper respiratory tract infection, and (2) the renal deposits of IgA consist of polymeric IgA1 and secretory IgA, which are mostly produced by the MALT [31,32,33]. Humans have two IgA isotypes. IgA1 accounts for 80%–85% of total serum IgA, which, at mucosal sites, is matched by local plasma cells (PCs) in the tonsils and nasal mucosa, whereas IgA2 usually predominates in the normal large bowel mucosa [34]. Intestinal bacteria cleave the amino acids in the hinge region of IgA1; in contrast, IgA2 is resistant to such cleavage by intestinal bacteria owing to the lack of 13 amino acids in its hinge region [35]. Consequently, the abundance of IgA2-producing PCs on the intestinal mucosal surface is considered to help efficiently defend against infection [35].

Interestingly, Sakai et al. showed that in patients with IgAN and chronic myeloblastic leukemia, bone marrow transplantation resulted in remission of IgAN as well as leukemia [36]. Furthermore, Imazawa et al. found that the transplantation of bone marrow stem cells from IgAN-onset mice to normal mice resulted in IgA deposition in their glomeruli, and that bone marrow transplantation from normal mice to IgAN-onset mice reduced their renal lesions, proteinuria, and serum IgA levels [37]. Suzuki et al. suggested that IgA-producing bone marrow cells in patients with IgAN may drive the development of IgAN [38]. On the basis of these findings, immunoregulatory impairment in the “mucosa–bone-marrow axis” has been hypothesized to occur in patients with IgAN [39]. 

The mucosal immune system is mainly divided into the GALT and NALT. However, it is unclear which sites significantly contribute to the production of Gd-IgA1 and the pathogenesis of IgAN.

## 4. Gut-Associated Lymphoid Tissue (GALT)

Patients with celiac disease or inflammatory bowel disease often develop IgAN, especially in Europe [40,41]. The unexpectedly high co-occurrence of IgAN with celiac disease, which is developed by intolerance to gluten, led to the proposal of a possible association between IgAN and intestinal disorder. One report indicated that a gluten-free diet reduced both gliadin-specific IgA and IgA-containing ICs and also altered the intestinal microbiota [42]. Furthermore, Papista et al. showed, in a double transgenic IgAN model mouse co-expressing human IgA1 and human CD89, that a gluten-free diet prevented the deposition of IgA and renal injury associated with IgAN, whereas the introduction of dietary gluten caused IgAN to develop [43]. These results suggest that digestive antigens, especially gluten, may be involved in the pathogenesis of IgAN [44].

A genome-wide association study recently revealed the role of gut microbial exposure in individuals at genetic risk of developing IgAN and found a relationship between IgAN and genes involved in immunity to gut pathogens [45]. Dietary and climate factors were related with the genetic risk; however, it was most strongly associated with regional pathogen diversity [45]. Most loci found to pose a risk for developing IgAN, such as ITGAM-ITGAX and CARD9, were related to inflammatory bowel disease as well as to maintaining the intestinal barrier and regulating the GALT against gut pathogens [45].

A targeted-release formulation of budesonide (TRF-budesonide) is designed to deliver budesonide to the distal ileum, a major site of mucosal B-cell localization within the GALT. Because this formulation is rapidly metabolized in the liver, which clears most of the drug, less than 10% of the drug is systemically effective, and it is, therefore, expected to have relatively fewer systemic side effects. In a clinical trial (named the NEFIGAN study), the TRF-budesonide group exhibited a significant decrease in the urinary protein creatinine ratio (UPCR), whereas the placebo group exhibited an increase in the UPCR after 9 months [46,47]. On the basis of this encouraging finding, a Phase 3, multicenter, randomized, double-blind, placebo-controlled trial is currently underway to confirm the safety and efficacy of TRF-budesonide. However, steroid-related adverse events were reported more frequently in the TRF-budesonide group; thus, the systemic effects of TRF-budesonide should be considered with caution.

B-cell-activating factor (BAFF) belonging to the tumor necrosis factor (TNF) family is a type 2 transmembrane protein belonging to the TNF superfamily of ligands, along with a proliferation-inducing ligand (APRIL) [48]. BAFF is produced by multiple cell types, such as dendritic cells and monocytes, and regulates the differentiation and survival of B cells by binding to receptors on these cells [49]. BAFF-transgenic (BAFF-Tg) mice exhibit a similar pathology to that of IgAN unless they are kept under germ-free conditions. In the absence of pre-existing commensal bacteria, following the colonization of germ-free BAFF-Tg mice with a limited commensal microbiota containing *Lactobacillus murinus*, IgA against *L*. *murinus* was produced, and IgAN was reconstituted in these colonized BAFF-Tg mice [50]. Therefore, commensal bacteria appear to be necessary for the development of IgAN in BAFF-Tg mice. However, the clinical trial of blisibimod, an anti-BAFF antibody, was discontinued in the phase 3 trial following the results of the phase 2 trial, and the role of BAFF in the pathogenesis of IgAN needs further verification.

IgA produced by intestinal tract plasma cells is secreted as a dimer or polymer linked by J-chain binding. This dimer or polymer then binds to the polymeric immunoglobulin receptor (pIgR), which is expressed on the basement membrane of mucosal epithelial cells and is secreted to the luminal side as secretory IgA [51]. However, inflammation, especially in inflammatory bowel disease, reportedly causes pIgR dysfunction. The secretion of IgA produced by the lamina propria to the mucosal surface is consequently inhibited, resulting in the transfer of this IgA into the blood instead. Patients with inflammatory bowel disease were found to have an elevated serum level of secretory IgA owing to pIgR dysfunction [52,53].

In other words, if the intestinal mucosa is constantly exposed to inflammation, such as in inflammatory bowel disease, antigen-specific IgA or mucosal IgA may migrate into the blood. However, most patients with persistent inflammatory bowel disease or gastrointestinal symptoms do not have IgAN, suggesting that IgA produced in the intestinal tract can migrate into the blood without causing secondary IgAN. Aizawa et al. demonstrated that bone marrow transplantation from IgAN-onset mice can experimentally reconstitute mouse IgAN, not only in normal mice but also in alymphoplasia mice that lack Peyer’s patches, lamina propria and all lymph nodes, doing so in a manner independent of IgA-producing-cell homing to secondary lymphoid tissues or mucosa [54]. This finding indicates that homing to the intestinal mucosa by the cells responsible for the production of aberrantly glycosylated IgA is not necessary.

Generally, specific antibodies against dietary proteins or gut microbiota members are not normally observed in serum, except in special circumstances when members of the gut microbiota enter the bloodstream. The involvement of gluten in IgAN has been demonstrated, particularly in Europe; however, a report from the United States of America indicated that the levels of antibodies to gliadin and TG2 (serological markers specific for celiac disease) did not differ between patients with IgAN and disease controls [55]. Thus, the involvement of celiac disease and gliadin in IgAN is still controversial. However, previous reports showed that from approximately one-third to one-half of patients with IgAN have an intestinal mucosa with sensitivity to gluten without the development of celiac disease [56,57]. In addition, anti-gliadin IgA antibodies were found to be correlated with high levels of ICs, and 71% of patients with IgAN showed a decrease in proteinuria and/or hematuria after adopting a gluten-free diet [58]. These findings together suggest that subclinical inflammation of the intestinal mucosa in response to antigens such as gluten may be involved in the pathogenesis of IgAN. Thus, it is necessary to elucidate the detailed mechanism by which antigen-specific IgA produced in the intestinal tract moves into the blood.

## 5. Nasal-Associated Lymphoid Tissue (NALT)

NALT is an immune-inducing tissue that consists mainly of the Waldeyer pharyngeal ring, composed of adenoids and palatine tonsils in humans. Regarding IgA subclasses, NALT-derived plasma cells produce significantly more IgA1 than IgA2, with a IgA1:IgA2 ratio of 9:1, whereas GALT-derived plasma cells produce IgA with a subtype ratio of 1:1 [34]. The effects of tonsillectomy (described in detail below) and the specific involvement of IgA1, rather than IgA2, in causing nephritis suggest that the main pathophysiology for IgAN is the homing of NALT-induced B cells to the bone marrow, centering on the tonsils.

To date, many reports have shown the efficacy of tonsillectomy in treating IgAN [59,60,61,62], and one nationwide multicenter cohort study in Japan found that, among patients with IgAN, undergoing a tonsillectomy was associated with a lower risk of renal outcomes [63]. In addition to these reports from Japan, a meta-analysis also provides credible evidence to support the use of tonsillectomy for patients with IgAN, especially those under long-term treatment [64], and the efficacy of tonsillectomy was also demonstrated in 98 Caucasian patients with IgAN in a Hungarian report [65].

Notably, the tonsils of patients with IgAN contain a significantly elevated proportion of IgA-positive cells and cells producing IgA polymers with J-chains [66,67]. It was found by mass spectrometry that the *O*-glycan structure of IgA1 produced by tonsillar lymphocytes was galactose-deficient in the hinge region [68]. Furthermore, the levels of gene expression of β1,3-galactosyltransferase (β3GalT), the polypeptide N-acetylgalactosaminyl-transferase 2, C1β3GalT-specific molecular chaperone, and Cosmc were significantly lower in CD19-positive B cells from the tonsils of patients with IgAN than in those from the tonsils of control volunteers [69]. These lower gene expression levels are related to the similar abnormal expression of glycosylation enzymes in IgA1-producing cell lines derived from patients with IgAN [19]. It has also been reported that, in patients with IgAN, the T-cell area in the tonsils is significantly expanded and the ratio of IgA/IgG-producing cells is high compared with that in patients with chronic tonsillitis. Furthermore, tonsillectomy performed at 1 year after kidney transplantation significantly decreased the level of serum Gd-IgA1 and the recurrence rate of histological IgAN after kidney transplantation [70]. On the bases of these findings, serum Gd-IgA1 derived from the tonsillar B cells likely plays an important role in the pathogenesis of IgAN.

There have been reports of the detection of specific exogenous antigens from pathogens such as methicillin-resistant *Staphylococcus aureus* and *Haemophilus parainfluenzae* in the glomeruli of patients with IgAN [71,72]. Consequently, it has been debated for many years whether specific antigens and IgA form ICs in the mucous membranes, blood, or kidneys, and if the IgA-containing ICs induce nephritis [73]. However, the reported antigen detection had low reproducibility in many cases; thus, it is not clear whether there is an exogenous-specific antigen involved in the pathogenesis of IgAN. Recently, the involvement of Toll-like receptors (TLRs), which are a group of central innate immune system receptors that recognize the common antigen structures of bacteria and viruses and work for host defense, has been suggested for IgAN. Activation of the innate immune system may increase the production of nephritic IgA, independently of specific antigens. Our research team established a grouped ddY (gddY) mouse model that develops IgA in the early period by selectively crossing ddY mice [74,75]. A genetic analysis revealed a strong correlation supporting the involvement of the gene for a TLR-signaling molecule (MyD88) in the development of IgAN. In addition, we confirmed that the expression of TLR9, which recognizes unmethylated microbial DNA as a ligand, is enhanced in the spleen of IgAN-onset mice [75]. Furthermore, nasal stimulation of these mice with the TLR9 ligand CpG-oligodeoxynucleotide (CpG-ODN) exacerbated renal injury and increased the levels of serum and mesangial IgA [76]. In human studies, we found that two SNPs in the TLR9 gene (rs352139 and rs352140) may be associated with IgAN histologic severity [76]. Additionally, tonsillar TLR9 and TLR9 SNP expression levels correlated with the efficacy of tonsillectomy with steroid pulse therapy in patients with IgAN [77]. Furthermore, the group in which serum Gd-IgA1 levels decreased after tonsillectomy alone exhibited significantly higher levels of tonsillar TLR9 expression and increased improvements in hematuria immediately after tonsillectomy compared with the group in which Gd-IgA1 levels only decreased after the addition of steroid pulse therapy after tonsillectomy. The results of these studies indicate that the Gd-IgA1-producing cells are present in palatine tonsils [78]. Recently, a genome-wide association study revealed that a *TNFSF13* (APRIL) variant is involved in IgAN susceptibility [79]. *TNFSF13* encodes a member of the TNF ligand family that is important to the IgA class-switch recombination and the development and survival of B cells [80,81]. Encouragingly, the use of an anti-APRIL antibody in IgAN model mice suppressed the levels of IgA and IgG and decreased the level urinary protein, and clinical trials assessing this antibody treatment are currently underway [82,83].

Muto et al. found that tonsillar germinal centers (GCs) of patients with IgAN included abnormal APRIL-producing cells, contributing to a significant upregulation of overall APRIL expression in the tonsils, in contrast with the GCs of control patients with tonsillitis. Furthermore, the aberrant APRIL expression in tonsillar GCs was positively associated with proteinuria, and those with an overexpression of aberrant APRIL in their tonsillar GCs were more likely to respond beneficially to tonsillectomy with a decrease in their levels of serum Gd-IgA1 [84]. Notably, the stimulation of tonsillar mononuclear cells with CpG-ODN increased the levels of BAFF and interferon-γ production by these cells in patients with IgAN, and tonsil cell BAFF expression was also elevated in patients with IgAN as compared that in patients with chronic tonsillitis [85]. Thus, IgAN may also develop owing to the overexpression of BAFF molecules on tonsil cells. Increased serum APRIL and BAFF levels in patients with IgAN and their correlation with prognosis suggest that the activation of APRIL/BAFF, mainly in the tonsils, contributes to the development and progression of IgAN [50,86].

## 6. The Difference between the NALT and GALT

Interestingly, despite being structurally and functionally very similar, Peyer’s patches and the NALT have completely different origin and developmental processes. Peyer’s patches begin to form during the embryonic stage of development and are completed at birth, whereas NALT formation begins postnatally [11,87]. In general, Peyer’s patch-targeted immunization induces a protective immunity that is broadly effective against antigens in the GALT, whereas NALT-targeted immunization efficiently generates antigen-specific immunity, mainly in the respiratory tract [12].

Plasmablasts derived from a mucosal immune response can be detected in the blood [88]. Interestingly, a recent report showed that gut-derived IgA^+^ plasmablast and/or plasma cells play an important role in dampening neuroinflammation in mice with experimental autoimmune encephalomyelitis [89]. Previously, basic homing of IgA-producing B cells has been reported [90]. The homing of B cells to the mucosa and peripheral tissues is fundamentally dependent on a unique combination of adhesion molecules and chemokine receptors; these expression patterns differ between IgA-producing B cells stimulated in the NALT and those stimulated in the GALT [90]. Over the course of the IgA plasmablast differentiation in the intestinal lymphoid tissue, CC Chemokine Receptor (CCR) 9 and α4β7 upregulation directs their homing to locations in the small intestine expressing C-C motif chemokine ligand (CCL) 25 and mucosal-addressing cell adhesion molecule (MAdCAM)-1 [90]. Thus, in general, plasmablasts induced by the GALT have difficulty migrating to the systemic immune system owing to its unique combination of adhesion molecules and chemokine receptor expression.

**Figure 2:**
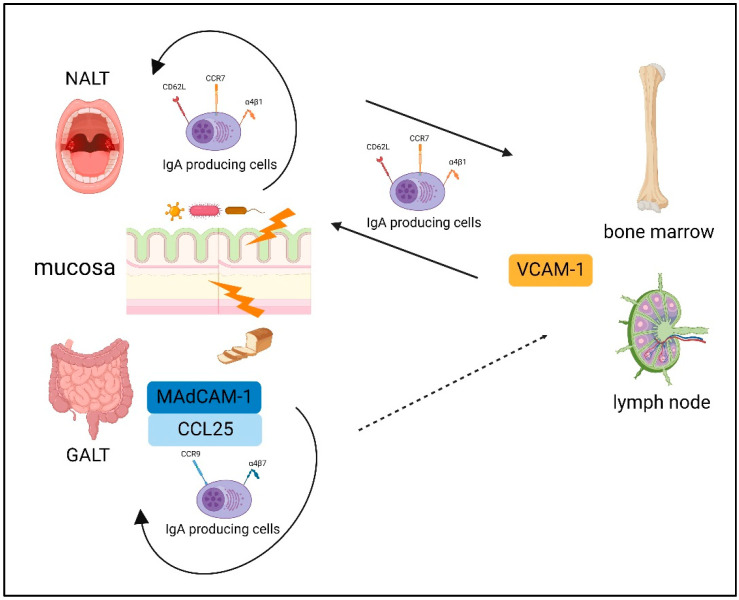
The basic dichotomy of B-cells homing. The expression patterns differ between IgA-producing B cells stimulated in the NALT and those stimulated in the GALT. The GALT induced CC Chemokine Receptor (CCR) 9 and α4β7 upregulation, which directs their homing to locations in the small intestine expressing mucosal-addressing cell adhesion molecule (MAdCAM)-1 and C-C motif chemokine ligand (CCL) 25. NALT induces the expression of α4β1, which interacts with vascular cell adhesion molecule (VCAM)-1, and the molecules l-selectin (CD62L) and CCR7, resulting in systemic homing. Created with BioRender.com.

The NALT also induces the expression of α4β1, which interacts with vascular cell adhesion molecule (VCAM)-1, and the molecules l-selectin (CD62L) and CCR7, resulting in systemic homing [91]. This probably reflects that the NALT acts as a “cross-road” between mucosal and systemic immunity [92]. The basic dichotomy of B-cells homing is summarized in Figure 2.

A recent report revealed that patients with IgAN showed increased levels of Gd-IgA1^+^ λ^+^ B cells expressing CCR10 and CCR9 [93]. However, it is unclear how NALT- and GALT-mediated integrin and chemokine receptors interactions in IgA-producing cells are involved in the pathogenesis of IgAN. Thus, further basic and clinical studies are required.

A recent survey demonstrated obvious differences between nations in their populations’ frequencies of intestinal complications, such as celiac disease and intestinal bowel disease, which were more frequent in European patients with IgAN [94]. In contrast, the frequencies of gross hematuria related with upper respiratory tract infections are similar according to the same survey, thus highlighting the pathogenic role of the NALT in patients with IgAN [94].

There are many studies, mainly from Japan, that have reported a beneficial effect of tonsillectomy on IgAN; however, some European studies have not found favorable results for this IgAN treatment [95,96,97]. Currently, the KDIGO guidelines suggest that tonsillectomy should not be performed as a treatment for IgAN, at least in Caucasian patients [98]. However, a favorable effect of tonsillectomy for treating IgAN was confirmed by a recent meta-analysis of studies that were mainly conducted in Asia. [99]. The studies in European populations that concluded that tonsillectomy may not be effective have some limitations. First, the number of patients who received a tonsillectomy after renal biopsy was limited to only 17 patients in the VALIGA study [95], and Piccoli et al. analyzed only 15 patients with IgAN who received tonsillectomy [96]. Although tonsillectomy is more effective in the early stages of IgAN, the German study on this topic investigated only the IgAN patients with progressed kidney disease (serum creatinine level was over 2 mg/dL) [97]. However, the results of a Hungarian study conducted on 98 Caucasian patients with IgAN supported the effectiveness of tonsillectomy [65]. Given these limitations and conflicting reports, we cannot conclude that tonsillectomy is ineffective for IgAN in Europeans, and the effectiveness of tonsillectomy should be stated with caution. To definitively determine the efficacy of tonsillectomy for IgAN, further basic and clinical studies are needed.

A recent study, conducted using biopsy samples from patients with IgAN, revealed that there are CD19-positive B cells that co-localize with IgA in the kidney [100]. Additionally, Currie et al. identified an elevated prevalence of genus *Neisseria* carriage in the tonsils and increased presence of serum anti-*Neisseria* IgA in patients with IgAN [101]. Moreover, after a nasal infection with *Neisseria* in BAFF-Tg mice, the serum levels of anti-*Neisseria* IgA were significantly increased, and anti-*Neisseria* IgA-secreting cells were found in the kidneys. This finding indicates that IgA-producing cells induced by exogenous antigen exposure in the respiratory tract can move to the kidney in the pathogenesis of IgAN [101]. Further studies are needed to determine the role of the specific IgA-producing cells localized in the kidney and the mechanisms that are responsible for promoting the migration of these cells from the airway to the kidneys.

## 7. Conclusions

According to a recent systematic review, IgAN has an incidence of at least 2.5 per 100,000 adults globally [102]. However, its prevalence varies geographically, and the prevalence of IgAN is much higher in East Asia than in North America or Europe [102,103]. There are many reports, mainly in Japan, regarding the beneficial effect of tonsillectomy on IgAN; however, some European studies have not found favorable results supporting the use of tonsillectomy for treating IgAN. Furthermore, in Europe IgAN is often associated with intestinal bowel disease and celiac disease, whereas this is rarely the case in Japan.

IgAN is the most frequent biopsy-proven primary glomerular nephritis, and is simply diagnosed by the deposition of IgA in mesangial region. Given that the epidemiologic heterogeneity of this disease was shown to depend on race and sex, there is a possibility that the molecular mechanisms responsible for its pathogenesis differ between Europe and Asia. Further basic and clinical studies are needed to clarify the mechanisms of IgAN pathogenesis.
